# The identification of probable sarcopenia in early old age based on the SARC-F tool and clinical suspicion: findings from the 1946 British birth cohort

**DOI:** 10.1007/s41999-020-00310-5

**Published:** 2020-03-19

**Authors:** R. M. Dodds, J. C. Murray, S. M. Robinson, A. A. Sayer

**Affiliations:** 1 AGE Research Group, Newcastle University Institute for Translational and Clinical Research, Newcastle upon Tyne, UK; 2 NIHR Newcastle Biomedical Research Centre, Newcastle University and Newcastle upon Tyne NHS Foundation Trust, 3rd Floor Biomedical Research Building, Campus for Ageing and Vitality, Newcastle upon Tyne, NE4 5PL UK

**Keywords:** Grip strength, Older people, EWGSOP2, Sarcopenia

## Abstract

**Purpose:**

The European Working Group on Sarcopenia in Older People 2 (EWGSOP2) consensus definition introduced the concept of probable sarcopenia as a basis on which to begin treatment. Our aims were to describe the prevalence of probable sarcopenia in older adults and to investigate the utility of (1) the SARC-F tool and (2) clinical risk factors for the identification of those likely to have probable sarcopenia.

**Methods:**

We used data from the 1946 British birth cohort at age 69, with 1686 participants included in the analyses. We used the EWGSOP2 cut points for weak grip strength and slow chair rise time, with the presence of one or both indicating probable sarcopenia. We examined the sensitivity and specificity of the SARC-F tool for probable sarcopenia. We also examined associations between clinical risk factors and probable sarcopenia.

**Results:**

The prevalence of probable sarcopenia was 19%. A SARC-F score of ≥ 4 had low sensitivity (15%) and high specificity (99%) for probable sarcopenia, whereas a score of ≥ 1 had higher sensitivity (65%) and reasonable specificity (72%). Three clinical risk factors were independently associated with probable sarcopenia: polypharmacy [OR 2.7 (95% CI 1.7, 4.2)], lower body osteoarthritis [OR 1.8 (95% CI 1.3, 2.6)] and physical inactivity [OR of 2.1 (95% CI 1.5, 2.8)].

**Conclusion:**

We have shown that EWGSOP2 probable sarcopenia is common in community-dwelling adults in early old age. Those with any positive responses to the questions in the SARC-F tool, a history of polypharmacy, lower body osteoarthritis or physical inactivity should be prioritised for the assessment of muscle strength.

**Electronic supplementary material:**

The online version of this article (10.1007/s41999-020-00310-5) contains supplementary material, which is available to authorized users.

## Key summary points

**Aim:**

To describe the prevalence of probable sarcopenia in a sample of older adults and to investigate (1) the SARC-F tool and (2) clinical risk factors in the identification of probable sarcopenia.

**Findings:**

The prevalence of probable sarcopenia at age 69 was 19%, and a SARC-F score of ≥ 1 had a reasonable balance of sensitivity (65%) and specificity (72%) for probable sarcopenia. Three clinical risk factors were independently associated with probable sarcopenia: polypharmacy, lower body osteoarthritis and physical inactivity.

**Message:**

Those with any positive responses to the questions in the SARC-F tool, a history of polypharmacy, lower body osteoarthritis or physical inactivity should be prioritised for the assessment of muscle strength.

**Electronic supplementary material:**

The online version of this article (10.1007/s41999-020-00310-5) contains supplementary material, which is available to authorized users.

## Introduction

Sarcopenia, the accelerated loss of muscle strength and mass, is linked to a range of adverse outcomes including disability [1, 2] and is amenable to interventions including resistance exercise training and nutritional supplementation [3, 4]. The recent European Working Group on Sarcopenia in Older People 2 (EWGSOP2) consensus definition facilitates the clinical identification of sarcopenia by incorporating the SARC-F tool as a screening measure and by introducing the concept of probable sarcopenia (PS) [5]. PS is the presence of low muscle strength based on poor performance in the grip strength test, the chair rise test or both. It is a basis on which to begin intervention, including in situations where it is not possible to assess muscle mass.

The sensitivity and specificity of the SARC-F tool to screen for sarcopenia defined according to the original EWGSOP consensus definition have been summarised in a systematic review and meta-analysis of seven studies [6]. It was found to have low sensitivity [0.21, 95% confidence interval (CI) 0.13–0.31], but high specificity (0.90, 95% CI 0.83–0.94). A subsequent study using a Turkish sample found similar values [7]. The latter study also found that the SARC-F tool had only a moderate sensitivity for detecting poor performance in the grip strength and chair rise tests, although using different cut points to those now recommended by EWGSOP2. A recent study of 306 community-dwelling participants at mean age 75 years found a sensitivity of 0.47 and specificity of 0.87 for EWGSOP2-confirmed sarcopenia using SARC-F [8]. As far as we are aware, there has been little exploration of the sensitivity and specificity of SARC-F to screen specifically for PS as defined by EWGSOP2.

The alternative approach recommended for screening for PS in the EWGSOP2 definition is to use clinical suspicion. Previous studies (using the earlier EWGSOP definition of sarcopenia) suggest that risk factors include being underweight, a history of routine/manual occupation, lower physical activity levels and the presence of long-term conditions [9–11]. We are not aware of studies that have examined risk factors for EWGSOP2 PS, and hence, it is less clear who to prioritise for the assessment of muscle strength. Using data from a British birth cohort study, the Medical Research Council National Survey of Health and Development (NSHD) [12, 13], our aims were firstly to describe the prevalence of PS in a sample of older adults and secondly to investigate the utility of (1) the SARC-F tool and (2) clinical risk factors in the identification of those most likely to benefit from the assessment of muscle strength.

## Methods

### Participants

We used data from the NSHD, a socially stratified sample of 5362 singleton births in 1 week of March 1946 in mainland Britain that have been followed up 24 times across life, most recently in 2015 (at age 69) [12, 13]. At age 69, study members still alive and with a known current address in mainland Britain (*n* = 2698) were invited to have a home visit; 2149 (79.7%) completed a visit, 55 (2.0%) completed a postal questionnaire instead and 494 (18.3%) did not participate [13]. Of the original cohort, 1026 (19.1%) had died, 578 (10.8%) were living abroad, 22 (0.4%) asked for their participation to be restricted to postal contacts, 621 (11.6%) had previously withdrawn from the study, and 417 (7.8%) had been lost to follow-up.

Ethical approval for this most recent follow-up was obtained from the NRES Queen Square Research Ethics Committee (14/LO/1073) and the Scotland A REC (14/SS/1009). Written, informed consent was obtained from study members for each component of the data collection.

### Assessment of muscle strength

Grip strength was assessed in the seated position using a Jamar Plus + Digital Hand dynamometer. Participants completed two trials in both hands, with the maximum value used in analyses. In the chair rise test, participants were asked to go from a seated position to standing with straight legs and back, and then sit down again, 10 times as fast as possible. We used a conversion equation to estimate the time taken to perform five chair rises, as described further in statistical analyses, below and in the supplementary methods.

We used the EWGSOP2 cut points for PS: grip strength of less than 27 kg in men and 16 kg in women, and/or time to complete five chair rises of greater than 15 s [5]. We considered those unable to do either test for health reasons to have PS for the purpose of analyses [14].

### Assessment of SARC-F and clinical risk factors

The SARC-F questionnaire has five components, comprising: difficulty in walking across a room, number of falls in the last year, strength (difficulty with lifting or carrying a 10 lb weight), difficulty with chair or bed transfers and difficulty with climbing stairs. Each is scored 0–2 in order of increasing difficulty and a score of 4 or more suggesting the presence of sarcopenia [15]. The difficulty in walking and fall components were asked in the NSHD as per the SARC-F questionnaire. The original strength component was not asked. We therefore used difficulty carrying shopping as 1 point and difficulty in holding a full kettle as 2 points. The chair and bed transfer component was assessed, but only in terms of difficulty with each of these two activities and not the degree to which this was present. We therefore scored difficulty with either chair or bed transfers as 1 point and difficulty with both as 2 points. Finally, difficulty with stair climbing was scored as 2 points for those unable to climb stairs, as well as those who reported that most or all of the time they needed to hold on, stop for a rest or go sideways or one step at a time. Those reporting less severe difficulty with stair climbing were scored as 1 point.

We chose a range of potential risk factors for PS. Body mass index (BMI) was calculated from height and weight measured using standard protocols and was grouped into below 25, 25–30 and above 30 kg/m^2^. Number of prescribed medications was categorised into 0, 1, 2–4, 5–8 (polypharmacy) and 9 + (excessive polypharmacy). Multimorbidity was based on the count of the following long-term conditions: asthma, cancer, diabetes, epilepsy, depression, high blood pressure, lung disease, transient ischaemic attack (TIA), eye disease, kidney disease, osteoarthritis, osteoporosis, Parkinson’s disease, rheumatoid arthritis and vascular disease (defined as having had angina, a heart attack, aortic aneurysm, raised cholesterol, deep vein thrombosis, atrial fibrillation, narrowing of arteries or pulmonary embolism). The count was divided into three categories: 0, 1 and 2 + conditions. Lower limb osteoarthritis, of particular interest given its association with poor performance in the chair rise test [16], was considered as a separate risk factor.

Lifestyle factors included smoking status (classified as never, ex-smoker or current), the frequency of current alcohol intake (with five categories from never to four or more times a week), regular fruit and vegetable consumption (daily or most days of the week) and participation in physical activity (sports or vigorous leisure activities in the last 4 weeks). Finally, occupation class at age 53 was categorised using the Registrar General’s Social Classification into three groups: I or II (high); IIINM or IIIM (medium); IV or V (low).

### Statistical analyses

To estimate the time taken to complete five chair rises based on the observed values for 10 rises assessed in NSHD, we used data from the second wave of the English Longitudinal Study of Ageing [17, 18]. Full details are provided in the supplementary methods section. In summary, a subset of participants of similar age to the present study performed both five and 10 chair rise tests as part of the same assessment, allowing us to produce a linear regression equation to estimate the time taken for five chair rises as used in EWGSOP2 [19].

We restricted our analytical sample to those with complete data on PS, SARC-F and risk factors. We calculated the sensitivity and specificity of different SARC-F cut points of for the identification of PS. We used multivariable logistic regression to examine independent risk factors for PS. We also repeated the analysis using Poisson regression, given the PS was not a rare (< 10%) outcome in this study [20]. The results were unchanged when we did this. We also repeated the logistic regression model with SARC-F positive participants excluded, in order to model the situation where clinical suspicion was being applied to those who were negative on SARC-F. We performed all analyses using Stata version 14.0 [21].

## Results

### Characteristics of the sample

A total of 1686 participants (51% female) aged 69–70 had complete data on PS, SARC-F score and clinical risk factors. As shown in the first column of Table 1, the majority were in the overweight range (with a mean BMI of 28.0 kg/m^2^), were taking between two and four prescribed medications and had none or one of the long-term conditions that were used to derive a measure of multimorbidity.

**Table 1:** Characteristics of sample

Characteristic	Whole sample (*N* = 1686)	Probable sarcopenia (*N* = 328)	No probable sarcopenia (*N* = 1358)
Sex			
Male	824 (48.9%)	147 (44.8%)	677 (49.9%)
Female	862 (51.1%)	181 (55.2%)	681 (50.1%)
BMI			
< 25	490 (29.1%)	71 (21.6%)	419 (30.9%)
25–30	702 (41.6%)	120 (36.6%)	582 (42.9%)
> 30	494 (29.3%)	137 (41.8%)	357 (26.3%)
Medications			
No medications	358 (21.2%)	40 (12.2%)	318 (23.4%)
Monopharmacy	278 (16.5%)	39 (11.9%)	239 (17.6%)
2–4 medications	698 (41.4%)	117 (35.7%)	581 (42.8%)
Polypharmacy (5–8 medications)	289 (17.1%)	100 (30.5%)	189 (13.9%)
Excessive polypharmacy (9 + medications)	63 (3.7%)	32 (9.8%)	31 (2.3%)
Long-term conditions			
None	596 (35.3%)	76 (23.2%)	520 (38.3%)
1	584 (34.6%)	108 (32.9%)	476 (35.1%)
2 or more	506 (30%)	144 (43.9%)	362 (26.7%)
Lower body osteoarthritis			
No	1418 (84.1%)	236 (72%)	1182 (87%)
Yes	268 (15.9%)	92 (28%)	176 (13%)
Occupation class			
IV or V (low)	200 (11.9%)	41 (12.5%)	159 (11.7%)
III (medium)	655 (38.8%)	145 (44.2%)	510 (37.6%)
I or II (high)	831 (49.3%)	142 (43.3%)	689 (50.7%)
Smoker status			
Current smoker	131 (7.8%)	33 (10.1%)	98 (7.2%)
Ex-smoker	1039 (61.6%)	209 (63.7%)	830 (61.1%)
Never smoker	516 (30.6%)	86 (26.2%)	430 (31.7%)
Alcohol intake			
Never, but have drunk alcohol in the past	159 (9.4%)	44 (13.4%)	115 (8.5%)
Monthly or less	275 (16.3%)	70 (21.3%)	205 (15.1%)
Two to four times per month	300 (17.8%)	54 (16.5%)	246 (18.1%)
Two to three times per week	436 (25.9%)	66 (20.1%)	370 (27.2%)
Four or more times a week	516 (30.6%)	94 (28.7%)	422 (31.1%)
Fruit and vegetable consumption			
Infrequent	584 (34.6%)	135 (41.2%)	449 (33.1%)
Daily or most days	1102 (65.4%)	193 (58.8%)	909 (66.9%)
Physical activity			
Inactive	954 (56.6%)	245 (74.7%)	709 (52.2%)
Active	732 (43.4%)	83 (25.3%)	649 (47.8%)

#### Muscle strength

Fourteen participants (< 1%) were unable to do the grip strength test due to health reasons and were assumed to have low muscle strength in later analyses. Mean (SD) measured grip strength was 40.5 (8.4) kg and 24.5 (5.8) kg in men and women, respectively.

Sixty-seven participants (4%) were unable to do the chair rise test due to health reasons and were assumed to have low muscle strength. A further 25 participants could only do between one and nine chair rises. Those who did fewer than five rises, five rises taking longer than 15 s or between six and nine rises with a time equivalent to performing five rises in greater than 15 s were also assumed to have low muscle strength. The median (IQR) time to complete five chair rises (calculated from the time taken to perform 10 rises, as described in the Supplementary Methods section) was 10.9 (9.2, 12.7) s, with no sex difference observed.

In total, 74 (4%) participants were unable to do one or both muscle strength tests due to health reasons. Compared to those who completed both tests, those who did not were more likely to have: a BMI in the obese category (47% compared to 28%, *P* = 0.001), lower body osteoarthritis (35% compared to 15%, *P* < 0.001) and two or more long-term conditions (46% compared to 29%, *P* = 0.002).

### Prevalence of probable sarcopenia

**Fig. 1:**
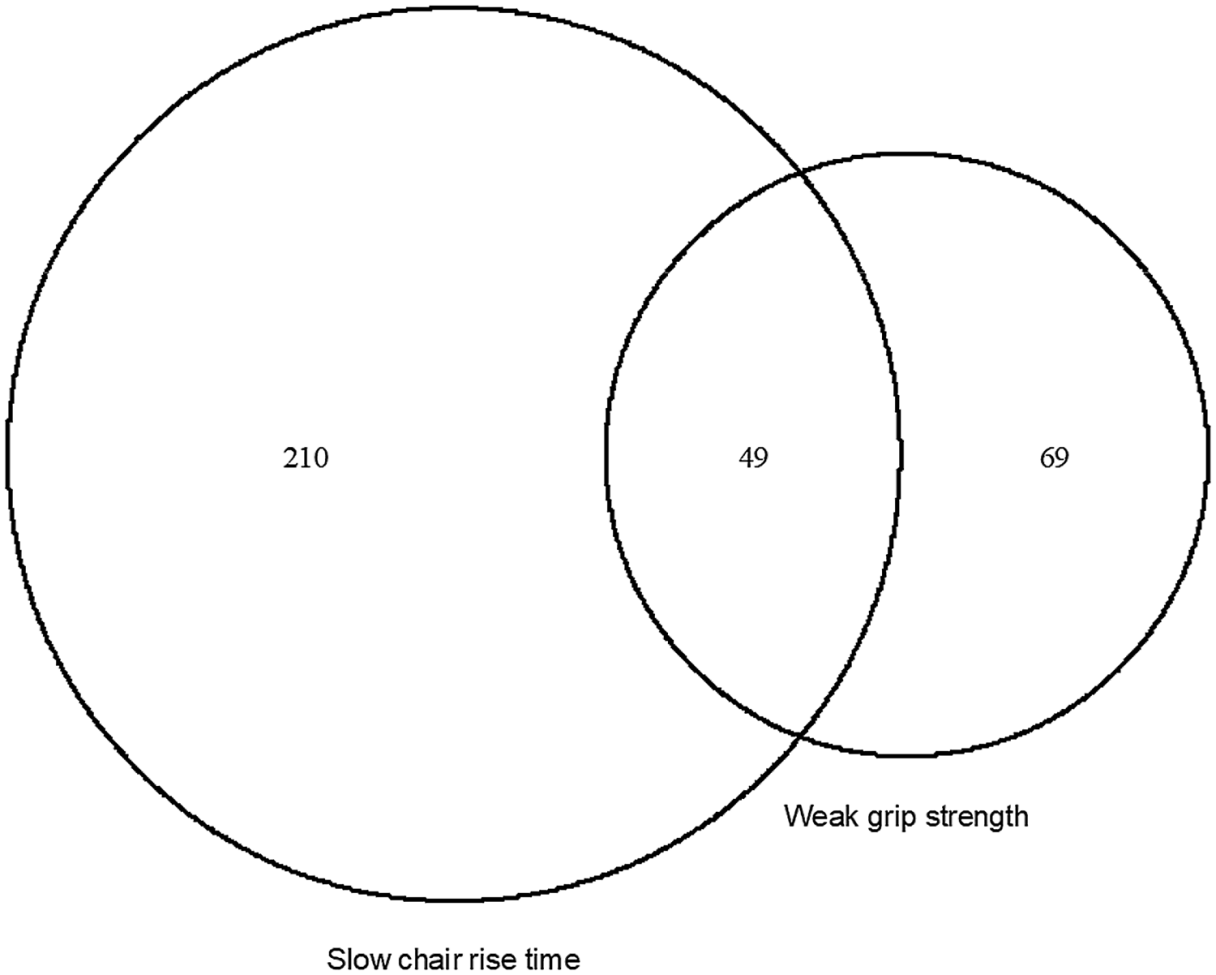
Overlap between different tests of muscle strength

Probable sarcopenia, defined as weak grip strength, slow chair rise time, or both, was present in 328 (19%) participants. Weak grip strength was present in 118 (7%) participants, including 14 who were unable to complete the test due to health reasons. Slow chair rise time was present in 259 (15%) participants, including 67 who were unable to complete the test due to health reasons. There was only limited overlap between weak grip strength and slow chair rise time, with 48 participants, or 15% of those with PS, having both (as shown in Fig. 1).

### The identification of probable sarcopenia

#### The SARC-F tool

**Fig. 2:**
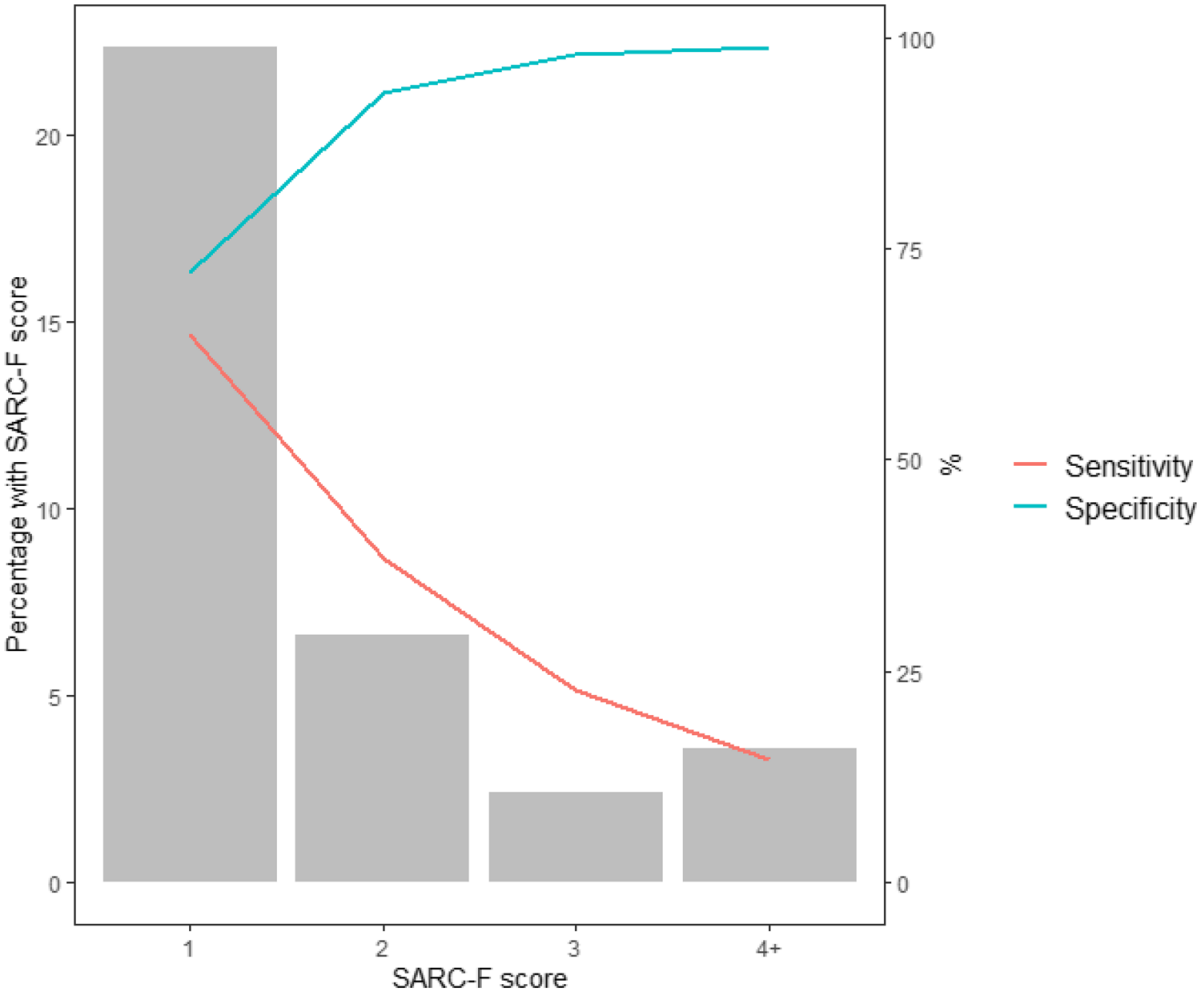
Prevalence of each SARC-F score and corresponding sensitivity and specificity for probable sarcopenia. The bars (left-hand *Y* axis) show the prevalence of each SARC-F score within the sample. The lines (right-hand *Y* axis) show the sensitivity and specificity of using each SARC-F score as a cut point for the identification of PS

The prevalence of any difficulty varied across the five components of the SARC-F tool: strength (7%), assistance walking (2%), transfer from a chair or bed (5%), climbing stairs (17%) and falls (20%). A positive (greater than or equal to four) SARC-F score was present in only 60 (4%) participants. A positive score was more likely in those with PS compared to those without (15% compared to 1%, *P *< 0.001). The sensitivity of a positive SARC-F score for PS was low at 15% with a specificity of 99%. However, a SARC-F cut point of one or above gave higher sensitivity (65%) and maintained reasonable specificity (72%), as shown in Fig. 2.

#### Clinical risk factors

As shown in Table 1, PS was more common in women and those with obesity, polypharmacy or excessive polypharmacy, two or more long-term conditions, lower body osteoarthritis, current or previous smoking, no or infrequent alcohol consumption, low or medium occupational class, infrequent consumption of fruit and vegetables, and physical inactivity. All but sex and smoking history reached statistical significance (*P* < 0.05) in univariable analyses. We also examined the characteristics of those with weak grip strength and slow chair rise time separately, as shown in Table 1 in the supplementary results section. We saw an overall similar pattern of characteristics for those with probable sarcopenia (that is poor performance in either or both of the two tests), although those with slow chair rise time appeared to have an even greater proportion of those with obesity and physical inactivity than those with weak grip strength. In supplementary results Table 1, we also examined the characteristics of those with both weak grip strength and slow chair rise time. This small group (*n* = 49) appeared to have a greater proportion of female participants and lower body osteoarthritis, compared to those with weak grip strength or slow chair rise time alone.

In a multivariable logistic regression model, number of medications, lower body osteoarthritis and physical inactivity continued to have a statistically significant association with PS, as shown in Table 2. These factors were independently associated with an approximate doubling of the odds of PS: polypharmacy (including excessive polypharmacy) compared to no medications had an OR (odds ratio) of 2.7 [95% CI (confidence interval) 1.7, 4.2]; lower body osteoarthritis had an OR of 1.8 (95% CI 1.3, 2.6); and physical inactivity had an OR of 2.1 (95% CI 1.5, 2.8). These associations were unchanged when the model was repeated with SARC-F positive participants excluded (results not shown).

**Table 2:** Multivariable model of risk factors for probable sarcopenia

Risk factor	Association between probable sarcopenia and risk factor shown		
	Odds ratio	95% CI	*P*
BMI category			0.071
< 25	Reference		
25–30	1.02	0.73–1.44	
> 30	1.41	0.99–2.01	
Number of medications			< 0.001
No medications	Reference		
Monopharmacy	1.08	0.66–1.76	
2–4 medications	1.22	0.82–1.84	
Polypharmacy (5–8 medications)	2.40	1.54–3.79	
Excessive polypharmacy (9 + medications)	4.42	2.31–8.49	
Long-term conditions			0.225
0	Reference		
1	1.25	0.89–1.76	
2 +	1.37	0.95–1.99	
Smoker status			0.638
Never smoker	Reference		
Current smoker	1.27	0.76–2.08	
Ex-smoker	1.10	0.81–1.48	
Alcohol consumption			0.275
Never, but have drunk alcohol in the past	Reference		
Monthly or less	1.01	0.63–1.63	
Two to four times per month	0.77	0.47–1.26	
Two to three times per week	0.67	0.42–1.08	
4 or more times a week	0.82	0.52–1.30	
Occupational class			0.360
I or II (high)	Reference		
IV or V (low)	0.81	0.52–1.23	
III (medium)	1.09	0.82–1.45	
Lower body osteoarthritis	1.89	1.35–2.65	< 0.001
Infrequent consumption of fruit and vegetables	1.10	0.83–1.44	0.510
Physical inactivity	2.06	1.55–2.77	< 0.001

*N* = 1686 in the model shown

*CI* confidence interval

## Discussion

We investigated the prevalence and identification of PS based on the EWGSOP2 definition in community-dwelling adults aged 69 participating in a British birth cohort study. We found a prevalence of 19% or approximately one in five participants. In terms of the components of PS, we found a higher prevalence of slow chair rise time (15%) compared to weak grip strength (7%). The SARC-F tool with a cut point of four or above, proposed for sarcopenia case finding, had high specificity (99%), but low sensitivity (15%), for PS. By comparison, a SARC-F cut point of one or above increased sensitivity (65%) whilst maintaining reasonable specificity (72%). Clinical risk factors which independently predicted the presence of PS were polypharmacy (five or more medications), lower body osteoarthritis and physical inactivity.

Few other studies have examined the prevalence of PS according to the EWGSOP2 definition. Kim and Won [22] reported a prevalence of 24% at mean age 76 using data from the Korean Frailty and Ageing Cohort Study; their findings and the present study highlight that in community-dwelling older people, there is a substantial proportion of individuals who would be classified as having PS and therefore require further assessment. In the original EWGSOP definition, the next step would be the assessment of muscle mass [23], although relevant imaging such as dual-energy X-ray absorptiometry may not always be feasible [24]. Under the EWGSOP2 definition, PS is now a basis on which to assess potential causes and begin treatment, even if measurement of muscle mass is not possible.

We recently found a prevalence of PS defined using weak grip strength in UK Biobank participants aged 60–70 of 8% [25], which is similar to the prevalence of weak grip strength in the present study (7%). In the present study, we saw limited agreement between weak grip strength and slow chair rise time, with only 15% of those with PS having poor performance on both tests. Further research to examine whether this limited agreement is also seen in other studies would be helpful. If confirmed, this would suggest that carrying out both tests in a clinical setting to assess for PS is warranted. This will require more time and carry the risk of older people not being able to complete both tests, especially the chair rise test [26]. However, there is evidence that inability to complete such tests is itself a marker or poor health [14] and therefore an important finding.

In terms of identification of those with PS, we found that a SARC-F cut point of four or more (as recommended in the EWGSOP2 definition) was uncommon in a community-dwelling sample at age 69, with a low sensitivity and high specificity for PS. A cut point of one or more had higher sensitivity whilst maintaining reasonable specificity. Difficulty with climbing stairs and falls in the last year were the most common SARC-F components reported by participants. The presence of either of these in a patient’s history could therefore act as a flag to needing assessment of PS.

The low to moderate sensitivity and high specificity of SARC-F as a screening tool for sarcopenia has been described previously [6, 8]. It has been suggested that the addition of calf circumference [27], age and BMI [28] may improve the diagnostic accuracy of the SARC-F tool for EWGSOP2 sarcopenia. SARC-F has also been shown to have higher sensitivity for EWGSOP2 sarcopenia in older patients with hip fracture [29], perhaps due to the higher prevalence of sarcopenia in this group (37%) compared to that typically seen in community-dwelling samples.

We also examined which clinical risk factors could be used to identify individuals with PS who require further assessment, equivalent to the concept of clinical suspicion described in the EWGSOP2 definition. Most factors tested were associated with PS, but those remaining independent in a multivariable analysis were polypharmacy, lower body osteoarthritis and physical inactivity. The first two of these can be assessed from a patient’s medical record, and the third can readily be assessed in a patient’s history. The next step would be to undertake a validation of these factors in a different sample including a wider age range.

We used data from a sample in early old age, and this may limit the generalisability of our findings. However, there is growing interest in the identification of sarcopenia in midlife and early old age, especially in the setting of patients with long-term conditions [25]. We also used a different question to assess self-reported strength than the one in the SARC-F tool (see “Methods” section), although given that most participants had an overall score of zero or one, this change would have been unlikely to cause a substantial reduction in the prevalence of participants who were SARC-F positive (a score of greater than or equal to four points).

In conclusion, we have shown that PS according to the EWGSOP2 definition is common in community-dwelling adults in early old age particularly if characterised by the presence of low grip strength or slow chair rise time. Our findings suggest that in this group, those with any positive responses to the questions in the SARC-F tool, a history of polypharmacy, lower body osteoarthritis or physical inactivity should be prioritised for the assessment of muscle strength. Future work should include the investigation of these relationships in clinical settings and at older ages.

## Electronic supplementary material

Below is the link to the electronic supplementary material.

Supplementary material 1 (DOCX 19 kb)
