# Supplementary material for: The identification of probable sarcopenia in early old age based on the SARC-F tool and clinical suspicion: findings from the 1946 British birth cohort
Source: Eur Geriatr Med. 2020 Mar 19;11(3):433–41. doi: 10.1007/s41999-020-00310-5 (PMC7280335; doi:10.1007/s41999-020-00310-5)
Supplement: Supplementary file 1 — Supplementary material 1 (DOCX 19 kb) [file 41999_2020_310_MOESM1_ESM.docx]

# Supplementary methods

## Estimation of time for five chair rises

The cut-off point for low muscle strength as measured using chair rises in the EWGSOP2 definition is set at > 15 seconds to complete five full rises [5]. The MRC National Survey of Health and Development (NSHD) used the time taken to complete 10 chair rises. We therefore used the approach previously taken by Cooper et al. [19] to estimate the time that would have been taken by participants to complete five rises. We used data from the second wave English Longitudinal Study of Ageing (ELSA) [17,18], a cohort study which measures the time taken for both five and 10 chair rises.

The second wave of ELSA contains 210 individuals (121 female) aged 69 with measurements of time taken for five and 10 chair rises. We used linear regression to estimate the relationship between these two times. We found no evidence of an interaction by sex.

We obtained the below equation relating the time taken to perform five rises (T_5_, seconds) from the time taken to perform 10 rises (T_10_, seconds). The $R^{2}$ for the model was satisfactory at 0.83.

$$T_{5}=0.79+0.44\times T_{10}$$

# Supplementary results

## Supplementary Table 1 Characteristics of those with weak grip strength, slow chair rise time, both weak grip strength and slow chair rise time

| **Characteristic** | **Whole Sample (N = 1686)** | **Weak grip strength or unable (N = 118)** | **Slow chair rise or unable (N = 259)** | **Both weak grip and slow chair rise (N = 49)** |
| --- | --- | --- | --- | --- |
| **Sex** |  |  |  |  |
| Male | 824 (48.9 %) | 50 (42.4 %) | 113 (43.6 %) | 16 (32.7 %) |
| Female | 862 (51.1 %) | 68 (57.6 %) | 146 (56.4 %) | 33 (67.3 %) |
| **BMI** |  |  |  |  |
| < 25 | 490 (29.1 %) | 35 (29.7 %) | 49 (18.9 %) | 13 (26.5 %) |
| 25 - 30 | 702 (41.6 %) | 43 (36.4 %) | 96 (37.1 %) | 19 (38.8 %) |
| > 30 | 494 (29.3 %) | 40 (33.9 %) | 114 (44 %) | 17 (34.7 %) |
| **Medications** |  |  |  |  |
| No Medications | 358 (21.2 %) | 14 (11.9 %) | 29 (11.2 %) | 3 (6.1 %) |
| Monopharmacy | 278 (16.5 %) | 16 (13.6 %) | 30 (11.6 %) | 7 (14.3 %) |
| Two to Four Medications | 698 (41.4 %) | 44 (37.3 %) | 90 (34.7 %) | 17 (34.7 %) |
| Polypharmacy | 289 (17.1 %) | 30 (25.4 %) | 83 (32 %) | 13 (26.5 %) |
| Excessive Polypharmacy | 63 (3.7 %) | 14 (11.9 %) | 27 (10.4 %) | 9 (18.4 %) |
| **Long term conditions** |  |  |  |  |
| 0 | 596 (35.3 %) | 24 (20.3 %) | 59 (22.8 %) | 7 (14.3 %) |
| 1 | 584 (34.6 %) | 39 (33.1 %) | 87 (33.6 %) | 18 (36.7 %) |
| 2+ | 506 (30 %) | 55 (46.6 %) | 113 (43.6 %) | 24 (49 %) |
| **Lower body osteoarthritis** |  |  |  |  |
| No | 1418 (84.1 %) | 86 (72.9 %) | 182 (70.3 %) | 32 (65.3 %) |
| Yes | 268 (15.9 %) | 32 (27.1 %) | 77 (29.7 %) | 17 (34.7 %) |
| **Occupation class** |  |  |  |  |
| IV or V (low) | 200 (11.9 %) | 15 (12.7 %) | 36 (13.9 %) | 10 (20.4 %) |
| III (medium) | 655 (38.8 %) | 51 (43.2 %) | 113 (43.6 %) | 19 (38.8 %) |
| I or II (high) | 831 (49.3 %) | 52 (44.1 %) | 110 (42.5 %) | 20 (40.8 %) |
| **Smoker status** |  |  |  |  |
| Current Smoker | 131 (7.8 %) | 13 (11 %) | 25 (9.7 %) | 5 (10.2 %) |
| Ex-smoker | 1039 (61.6 %) | 70 (59.3 %) | 168 (64.9 %) | 29 (59.2 %) |
| Never smoked | 516 (30.6 %) | 35 (29.7 %) | 66 (25.5 %) | 15 (30.6 %) |
| **Alcohol intake** |  |  |  |  |
| Never, but have drunk alcohol in the past | 159 (9.4 %) | 20 (16.9 %) | 34 (13.1 %) | 10 (20.4 %) |
| Monthly or less | 275 (16.3 %) | 24 (20.3 %) | 54 (20.8 %) | 8 (16.3 %) |
| Two to four times per month | 300 (17.8 %) | 13 (11 %) | 50 (19.3 %) | 9 (18.4 %) |
| Two to three times per week | 436 (25.9 %) | 28 (23.7 %) | 48 (18.5 %) | 10 (20.4 %) |
| 4 or more times a week | 516 (30.6 %) | 33 (28 %) | 73 (28.2 %) | 12 (24.5 %) |
| **Fruit & vegetable consumption** |  |  |  |  |
| Infrequent | 584 (34.6 %) | 53 (44.9 %) | 105 (40.5 %) | 23 (46.9 %) |
| Daily or most days | 1102 (65.4 %) | 65 (55.1 %) | 154 (59.5 %) | 26 (53.1 %) |
| **Physical activity** |  |  |  |  |
| Inactive | 954 (56.6 %) | 82 (69.5 %) | 201 (77.6 %) | 38 (77.6 %) |
| Active | 732 (43.4 %) | 36 (30.5 %) | 58 (22.4 %) | 11 (22.4 %) |
